# A CRISPR/cas13a-assisted precise and portable test for *Brucella* nucleic acid detection

**DOI:** 10.3389/fcimb.2025.1545953

**Published:** 2025-03-21

**Authors:** Haiwen Liu, Ling Xu, Ying Xiu, Na Ta, Qingqing Xu, Yu Fan, Kun Li, Hongyan Zhao, Dongri Piao, Feng Ren, Hai Jiang

**Affiliations:** ^1^ National Key Laboratory of Intelligent Tracking and Forecasting for Infectious Diseases, National Institute for Communicable Disease Control and Prevention, Chinese Center for Disease Control and Prevention, Beijing, China; ^2^ Ulanqab Center for Disease Control and Prevention, Department of Endemic Diseases and Brucellosis Prevention and Control, Jining, China; ^3^ Beijing Youan Hospital, Capital Medical University, Beijing Institute of Hepatology, Beijing, China; ^4^ Inner Mongolia Center for Animal Disease Control and Prevention, Department of Public Health, Inner Mongolia Autonomous Region Animal Disease Control Center, Inner Mongolia Autonomous Region, Hohhot, China; ^5^ Inner Mongolia Autonomous Region Center for Disease Control and Prevention, Department of Brucellosis, Inner Mongolia Autonomous Region, Hohhot, China

**Keywords:** *Brucella*_1_, brucellosis_2_, CRISPR-Cas13a_3_, fluorescence detection_4_, nucleic acid detection_5_, RAA_6_, strip test_7_, zoonosis_8_

## Abstract

**Introduction:**

*Brucella* infection in humans or animals can lead to brucellosis, which has the potential to significantly impact both the economy and public health. Currently, molecular biological methods for diagnosing brucellosis are either complex or have low sensitivity, and it is difficult to apply them in real-life settings in the field. Therefore, this study aims to establish a rapid and convenient nucleic acid-based molecular biology method for on-site rapid detection of *Brucella* and early clinical screening of brucellosis.

**Methods:**

Based on the conserved sequence of the *Brucella* Bcsp31 gene, we designed CRISPR RNA (crRNA) and RAA primers. We developed a fluorescence detection method and a paper strip detection method by integrating RAA amplification with CRISPR/Cas13a detection. We applied these methods to analyze 100 samples of suspected brucellosis-infected milk, 123 samples of human whole blood, and 100 samples of sheep vaginal swabs in order to validate their practical utility.

**Results:**

The RAA-CRISPR/Cas13a *Brucella* fluorescence detection method and the strip test method had detection limits of 10_0_ copies/μL and 10_1_ copies/μL, respectively, and both methods had a specificity of 100%. The positivity rate of the RAA-CRISPR/Cas13a fluorescence detection method for the milk, human whole blood, and sheep vaginal swab samples was 93% (93/100), 82.12% (101/123), and 91% (91/100), respectively; the strip test method, 87% (87/100), 64.23% (79/123), and 76% (76/100), respectively.

**Conclusion:**

In this study, we have developed a RAA-CRISPR detection method based on the *Brucella* BCSP31 gene, with potential applications in the identification of *Brucella* nucleic acid and implications for clinical diagnosis of brucellosis.

## 1 Introduction

The *Brucella* genus is a Gram-negative intracellular parasitic bacterium genus that comprises 12 species, including *B. melitensis* (sheep strain), *B. suis* (swine strain), *B. abortus* (cattle strain), *B. ovis* (sheep strain), *B. canis* (canine strain), and *B. neotomae* (wood rat strain), as well as species isolated from marine animals such as *B. ceti* and *B. pinnipedialis* (whale strain), the soil isolate *B. microti*, the breast implant material isolate *B. inopinata*, the red fox isolate *B. vulpis*, and the *B. papionis* (Kurmanov et al., 2022), with the cattle and sheep strains being the predominant pathogens causing disease in animals (Khoshnood et al., 2022). Brucellosis is a zoonotic infectious disease caused by *Brucella* infection that can result in allergic reactions in both humans and animals (Xiao et al., 2022). The prevalence of brucellosis varies across countries and regions worldwide, and it particularly prevalent in areas where the animal husbandry industry in well developed. *Brucella* has the ability to infect wild animals, livestock, and humans (Samadi et al., 2024).

*Brucella* is capable of entering the host organism through multiple pathways, including the skin mucosa, digestive tract, and respiratory tract (Sun et al., 2020; González-Espinoza et al., 2021). *Brucella* infection primarily results in reproductive system impairment and leads to stillbirths, miscarriages, and reduced milk production in female animals (Banai et al., 2021). Brucellosis can be transmitted to humans not only through the consumption of milk or undercooked meat from infected animals but also when humans have direct contact with animals afflicted with brucellosis (Dadar et al., 2019). In humans, the symptoms of *Brucella* infection include fever, diaphoresis, fatigue, arthralgia, and hepatosplenomegaly (Hasanjani Roushan et al., 2016; Zhang et al., 2022). In addition to being a threat to human health, the brucellosis epidemic has resulted in significant economic losses for the livestock industry and has severely impacted the development and economic viability of animal husbandry. The key to the prevention and control of brucellosis lies in the early detection and timely treatment or culling of infected animals to prevent the further spread of brucellosis (Dadar et al., 2021). However, most pastures and farms are located in the suburbs and less developed areas of northern China (where nomadic farming is practiced), and the local medical institutions or grassroots medical and health service centers often lack the manpower and financial resources to build first-class testing laboratories. Therefore, the choice of testing method is particularly important in grassroot areas with poor economic conditions that require large-scale screening. Importantly, while offering high sensitivity and specificity, simplicity, speed, and low cost, the ideal method should also be convenient for application in the field.

Currently, the diagnosis of brucellosis is based on the brucellosis diagnostic criteria WS269-2019, which include epidemiological history, clinical manifestations, and the results of laboratory tests for bacterial separation and serological detection methods. The laboratory diagnostic methods include bacterial isolation and culture, serological diagnosis, and nucleic acid-based molecular biological diagnosis (Yagupsky et al., 2019). Bacterial isolation and culture is considered the “gold standard” for diagnosing brucellosis, but it requires high-tech laboratory equipment and setup as well as a three-level biosafety laboratory (Di Bonaventura et al., 2021). In addition, bacterial cultivation is time consuming and has a low detection rate, and also poses a risk of infection to the laboratory staff (Song et al., 2021). Common diagnostic methods, such as Rose Bengal plate test (RBPT) (Vinetz et al., 2011), serum agglutination test (SAT) (Shemesh and Yagupsky, 2011), and other serological techniques, are commonly employed; however, their outcomes are heavily reliant on the subjective assessment of laboratory personnel and can, therefore, potentially result in false-positive or false-negative results (Avijgan et al., 2019). Nucleic acid-based molecular biological methods for *Brucella* diagnosis include real-time fluorescence quantitative PCR and ddPCR methods; however, they require large instruments and have a long amplification time (Whale et al., 2012; Zeybek et al., 2019). In addition, the operational process is complex and may generate aerosols, potentially contaminating the sample and generating false-positive results. Therefore, there is still a need to develop a method with high sensitivity, specificity, minimal equipment requirements, and the capability for rapid visual detection of *Brucella* in the field.

The CRISPR/Cas system is utilized by bacteria and archaea to defend against phages or foreign genetic material (Xu and Li, 2020). It a pivotal tool in molecular biology due to its high sensitivity and specificity for the detection of bacterial or viral nucleic acid. Some of the widely employed systems include CRISPR/Cas12 and CRISPR/Cas14 for DNA detection and CRISPR/Cas13 for RNA detection (Khan et al., 2019; Qin et al., 2019; Li et al., 2022). In contrast to PCR-based detection methods, the recently developed nucleic acid detection technology utilizing the CRISPR/Cas system does not require thermal cycling or intricate laboratory systems (Wang et al., 2020). Integrating biotin-labeled test strips with CRISPR/Cas detection technology could be the solution for rapid visual identification of *Brucella* in resource-constrained settings (Dang et al., 2023). Accordingly, this study presents a rapid and convenient visual detection test strip for *Brucella* nucleic acid based on RAA-CRISPR/Cas13a. The efficiency of this method was compared to that of a fluorescence-based RAA-CRISPR/Cas13a detection system and qPCR. Our method exhibited high sensitivity, specificity, and rapidity and was suitable for the detection of *Brucella* DNA in human whole blood, milk, and sheep vaginal swab samples.

## 2 Materials and methods

### 2.1 Sample collection

Hundred fresh milk samples were collected from a dairy farm located in Chaoyang District, Beijing, Gansu Province, Lanzhou City. Additionally, 100 vaginal swab samples were collected from a sheep farm located in Bayannaoer City, Inner Mongolia Autonomous Region, and 123 whole blood samples were collected from patients with suspected brucellosis at the Mongolian Medicine Research Institute of Tongliao City, Inner Mongolia Autonomous Region. We collected 4 mL of fasting venous blood from the 123 patients with suspected brucellosis. These samples were used for *Brucella* serological testing in accordance with the “Diagnosis for brucellosis” (WS269-2019). Suspected cases are defined as patients with an epidemiological history and corresponding clinical manifestations (such as persistent fever, sweating, fatigue, and muscle and joint pain). Confirmed cases are defined as suspected cases with SAT antibody titers of 1:100 (++) or isolation of *Brucella*. All collected samples were stored in a -80°C freezer for less than one month. In order to protect the patients’ privacy, patient-specific information was removed by assigning numbers to the patients and referring to the samples by the same numbers. This study has been approved by the Medical Ethics Committee of the Mongolian Medical Research Institute in Tongliao City, Inner Mongolia (Approval No.: TLSMYYJS-2023-1-004), and informed consent was obtained from patients and their relatives.

### 2.2 Plasmid synthesis and nucleic acid extraction

The BCSP31 gene (GenBank: MH045846.1) of *Brucella* was inserted into the pUC57 vector for the synthesis of the recombinant positive reference plasmid, which was subsequently constructed and purified by Biomed. The droplet digital PCR (ddPCR) instrument was employed to quantify the copy number of plasmid concentration. The primers and probe utilized were identical to those used in the quantitative PCR (qPCR) method. In this study, reference strains of *Brucella*, namely, 544A, 16M, and 1330S, were cultured and inactivated in a BSL-3 laboratory by trained laboratory personnel. The bacterial cultures were heat treated at 100°C for 60 min to ensure complete inactivation, and no viable bacteria were detected on culture. Nucleic acids from the bacterial suspension, blood samples, milk samples, and vaginal swab samples were extracted using the DNeasy Blood & Tissue Kit (Qiagen), and the extracted nucleic acids were stored at -20°C for future use.

### 2.3 Design and synthesis of crRNA

**Figure 1:**
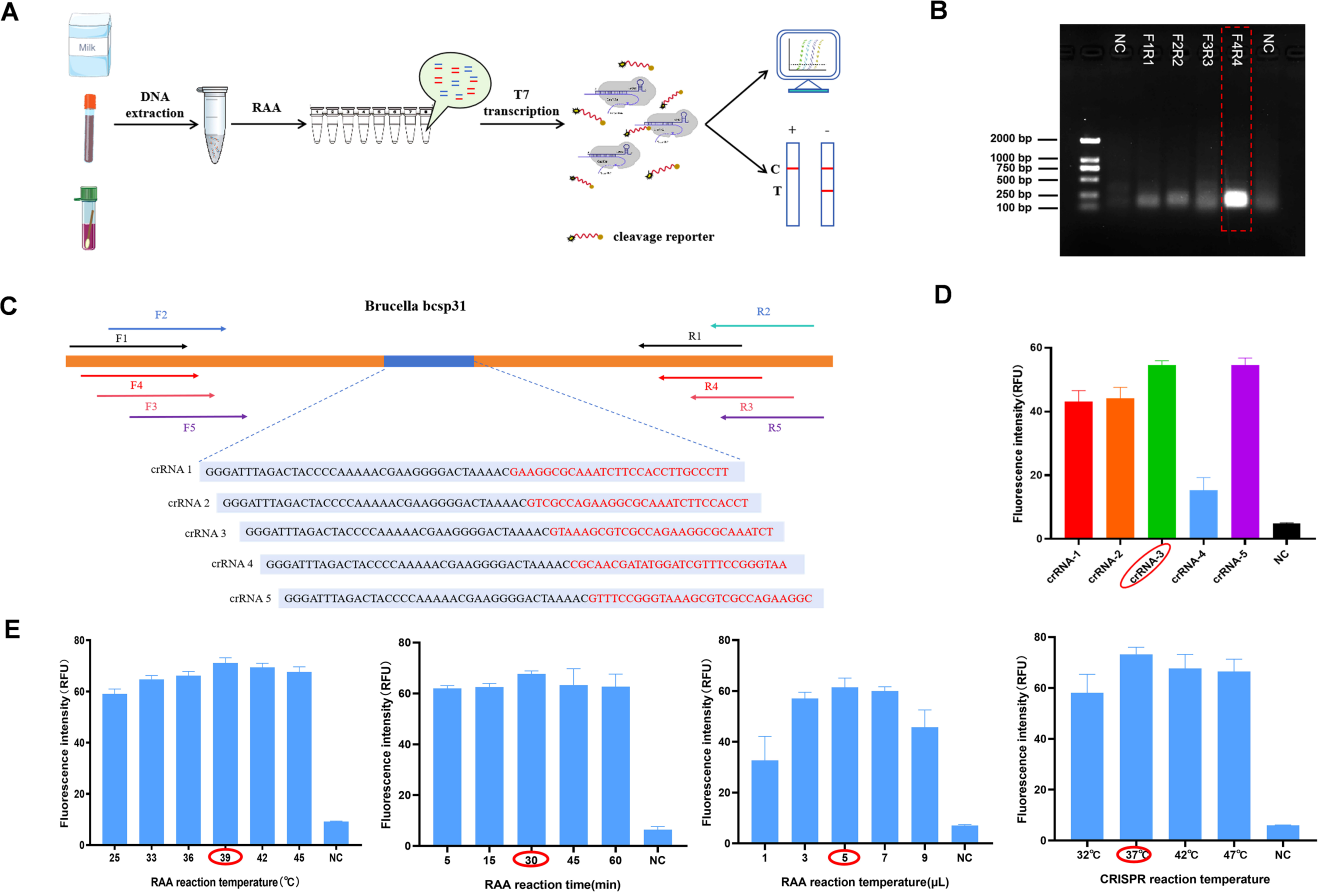
Construction of a Cas13a-crRNA Detection System for Brucella DNA **(A)** Protocol of the Cas13a-crRNA detection system for Brucella DNA detection. **(B)** The schematic diagram comprises 5 crRNAs and 4 pairs of RAA primers, with the bands (5′~3′) denoting the conserved regions within the screened HBV sequences. **(C)** Screening of RAA primers. The amplified Brucella-positive plasmid (105 copies/μL) obtained from RAA was analyzed using DNA agarose gel electrophoresis. The selected F4R4 primers were further validated with the Brucella-positive plasmid (105 copies/μL). **(D)** Screening of crRNA. The fluorescence values of various crRNA endpoints are presented and compared in the figure. RAA primers were utilized to amplify Brucella-positive plasmids (105 copies/μL) as templates for Cas13a-crRNA fluorescence detection, and five types of crRNA were validated. **(E)** Optimization of RAA reaction temperature (25°C, 33°C, 36°C, 39°C, 42°C, and 45°C), time (5, 15, 30, 45, and 60 min), and template volume (1, 3, 5, 7, and 9 μL). Fluorescence intensity corresponding to the CRISPR reaction temperatures 32°C, 37°C, 42°C, and 47°C.

The RAA primers and crRNA designed in this study were developed entirely independently, without the aid of any software. The crRNA consists of two components. One is a 28 - base - pair spacer region, which was designed based on the *Brucella* Bcsp31 gene and avoiding the RAA primer region. The other is a repeat sequence that binds to the Cas13 protein. (**Figure 1C**). Based on the designed crRNA sequence, single-stranded DNA was synthesized and subjected to PCR amplification using primers containing the T7 promoter sequence to generate the complete crRNA template. The crRNA template was mixed with T7 RNA polymerase and transcribed *in vitro* using the NEB HiScribe T7 Quick High-Yield RNA Synthesis Kit (New England Biolabs Inc.). The 20 μL reaction system consisted of 10 μL NTP mix, 2 μL T7 RNA polymerase, 3 μL crRNA-1 to 5, and 5 μL RNase-free water. Following overnight incubation at 37°C, the synthesized crRNA was purified using Sangon Biotech’s RNA Rapid Concentration and Purification Kit. (**Supplementary Tables 1 and 2**).

### 2.4 qPCR analysis

The primers and probe for qPCR were sourced from the literature (Probert et al., 2004). The forward and reverse primers for the qPCR reactions were 5′-GCTCGGTTGCCAATATCAATGC-3′ and 5′-GGGTAAAGCGTCGCCAGAAG-3′, respectively. A FAM-BHQ1-labelled probe was used for fluorescence detection, with the following sequence: 5′-AAATCTTCCACCTTGCCCTTGCCATCA-3′. The primers and probe were provided by Sangon Biological Engineering (Shanghai) Co. Ltd. The qPCR reaction system consisted of 10 μL of the PCR mix, 1.5 μL each of the forward and reverse primers and probe, 2.5 μL of sterile enzyme-free water, and 3 μL of the template DNA. The reaction conditions included a pre-denaturation step at 95°C for 3 min, followed by denaturation at 95°C for 15 s, annealing at 61°C for 30 s, and a total of 45 cycles, and extension at 40°C for an additional duration of 30 s.

### 2.5 RAA reactions

The amplification was carried out according to the instructions of the Hangzhou Public Test RAA amplification kit (basic type). According to the recommended reaction system, 25 μL A buffer, 13.5 μL water, and 2 μL upper and downstream primers (10 nM) were evenly mixed and added into an eight-strip tube containing lyophilized powder, and 5 μL sample DNA was added to the mixture. Next, 2.5 μL of Buffer B was added to the eight-strip tube cap, and the tube cap was closed. The octane discharge was centrifuged at the medium and low speed settings in a centrifuge for 15 s and then placed in a constant temperature metal bath at 39°C for 30 min.

### 2.6 Construction of the CRISPR detection system for *Brucella* DNA

The *Brucella abortus, Brucella melitensis*, and *Brucella suis* biotype I reference strains, that is, 544A, 16M, and 1330S, respectively, were cultured and inactivated by professional laboratory technicians in a biosafety level 3 laboratory to obtain bacterial broth. The bacteria were inactivated by heating at 100°C for 60 min. After culture, 500 µL of the inactivated bacterial solution was transferred to a 1.5-mL EP tube after ensuring that there was no viable bacterial growth. The blood samples used in this study were whole blood samples, and the milk samples and vaginal swab samples were centrifuged at high speed and then the precipitate was added with normal saline for use. The blood, milk, and vaginal swab samples were collected in accordance with the instructions of the DNeasy Blood & Tissue Kit for DNA extraction. During the nucleic acid extraction process of samples, the nucleic acids of all samples were extracted as soon as possible after collection. Before being tested, all the extracted nucleic acids were stored in a -20°C refrigerator to better ensure the accuracy of experimental data.

The extracted DNA was used as a template, and after RAA amplification, the amplified product was used as a CRISPR reaction template. The target single-stranded DNA obtained after the RAA amplification reaction was transcribed into ssRNA via T7 transcriptase. Next, the crRNA binds to ssRNA, and cas13a trans-cleavage activity is activated, which leads to the cleavage of RNA reporters. The cleaved RNA reporter can be detected by a fluorescence signal collection instrument or interpreted by a test strip (**Figure 1A**).

### 2.7 Fluorescence-based CRISPR–Cas13a detection

The CRISPR detection system consists of an RNase inhibitor (New England Biolabs Inc.), 1 μL LwCas13a, 1.5 μL crRNA, 2 μL NTP Mix (New England Biolabs Inc.), 0.5 μL of T7 RNA Polymerase (New England Biolabs Inc.), 0.5 μL of HEPES buffer solution (ThermoFisher Scientific Inc., USA), 0.25 μL MgCl_2_ solution (1 M), 2.5 μL quenched fluorescence RNA reporter (RNAse Alert; Thermo Scientific, Waltham, MA, USA), 5 μL of target nucleic acid, and 10.75 μL of RNase-free water. The reaction system was continuously collected at 37°C in a fluorescence signal collector (Roche LC480 real-time fluorescence quantitative PCR instrument) for 60 min, and the fluorescence signal was collected every 2 min for 30 cycles.

### 2.8 Lateral flow strip-based CRISPR–Cas13a detection

The test strip was constructed with a sample pad consisting of a combination of colloidal gold-labeled FAM antibody pads, avidin lines, FAM antibody lines, and an absorption mat. The sample liquid was dripped onto the test paper using the siphon effect and passed through the gasket line (T) and control line (C). Upon detection of the target nucleotide by the Cas13a-crRNA complex, Cas13a initiates trans-cleavage activity to cleave the reporter RNA (FAM-reporter RNA-biotin). This results in cracking of the reporter RNA and prevents colloidal gold molecules from remaining at line (T), which indicates a positive result. Conversely, formation of the “colloidal gold-labeled FAM antibody-RNA FAM report-avidin biotin” complex allows colloidal gold molecules to reside at test line (T), which indicates a negative result. The CRISPR detection system consists of 2 μL RNase inhibitor (New England Biolabs Inc.), 2 μL LwCas13a, 3 μL crRNA, 4 μL NTP Mix (New England Biolabs Inc.), 1 μL of T7 RNA Polymerase (New England Biolabs Inc.), 1 μL of HEPES buffer solution (ThermoFisher Scientific Inc., USA), 0.5 μL MgCl_2_ solution (1 M), 5 μL quenched fluorescence RNA reporter (5′-6-FAM/UUUUUUUUUUUUUUUUUUUU-Bio-3′), 5 μL of target nucleic acid, and 26.5 μL of RNase-free water. The reaction system, prepared with a volume of 50 μL, was incubated in a metal bath at 37°C for 30 min. Following the reaction, a volume of 45 μL of the product was transferred using a pipette onto a strip. After an additional 30-min incubation period, the results were read and recorded by capturing images.

### 2.9 Optimization of reaction conditions

The fluorescence signal data were analyzed by setting different reaction conditions with unique changes to screen for the best reaction conditions. Using *Brucella* recombinant positive plasmid at a concentration of 10^5^ copies/μL as the template, RAA was set at different reaction temperatures of 30°C, 33°C, 36°C, 39°C, 42°C, and 45°C, and different reaction times of 5 min, 15 min, 30 min, 45 min, and 60 min. The template volumes were also set to varying levels such as 2 µL, 4 µL, 6 µL, and 10 µL. For the RAA amplification reactions, different CRISPR reaction temperatures of 25°C, 28°C, 31°C, 37°C, 40°C, and 43°C were set to optimize the reaction conditions.

### 2.10 Assessment of detection capabilities

In order to verify the sensitivity of the established method, the reference strains of the recombinant positive plasmids of *B. abortus, B. melitensis*, and *B. suis* biotype I, namely, 544A, 16M, and 1330S, respectively, were diluted in gradient to obtain concentrations of 10^5^ copies/μL, 10^4^ copies/μL, 10^3^ copies/μL, 10^2^ copies/μL, 10^1^ copies/μL, 10^0^ copies/μL, and 10^-1^ copies/μL. The above concentration gradient was used as the template for RAA amplification. After the completion of the amplification reaction, 5 μL of the mixture was used as the template for subsequent CRISPR/Cas13a detection reaction, and CRISPR fluorescence detection and dip-strip detection were performed to determine the detection limits of the two detection methods. *Vibrio cholerae, Vibrio parahaemolyticus, Yersinia enterocolitica, Salmonella typhimurium*, and *Ochrobactrum anthropi* were selected to determine the specificity of the detection methods.

### 2.11 Statistical analysis

GraphPad Prism software version 8.0 (GraphPad Inc., La Jolla, CA, USA) was used to determine the means and SDs for analysis of variance (ANOVA). A paired *t*-test was used to detect the mean differences for each quantitative index. All statistical tests were two-sided, and statistical significance was set at P < 0.05.

## 3 Results

### 3.1 Optimization of conditions for the *Brucella* DNA CRISPR detection system

In order to enhance the detection of *Brucella* nucleic acid in actual test samples, we established methods to optimize the CRISPR/Cas13a system. We determined the optimal primer RAA reaction temperature, time, and template size, as well as the optimum CRISPR reaction temperature based the fluorescence signal intensity. To determine whether the filter condition was optimal, the strip method was used and the stripe color shades were assessed. The results of agarose gel electrophoresis showed that the fourth pair of primers (F4 and R4) had the highest amplification efficiency, and this was identified as the best primer pair for RAA (**Figure 1B**). crRNA-3 was selected as the best crRNA for the CRISPR reaction because it had high fluorescence intensity and the best amplification effect (**Figure 1D**). The results indicated that the optimal reaction temperature for RAA was 39°C; the optimal reaction time, 30 min; and the optimal template volume, 5 μL. With the fluorescence detection method, it was observed that the highest fluorescence intensity was recorded at 37°C (**Figure 1E**).

### 3.2 Capability of the CRISPR detection system for *Brucella* DNA

**Figure 2:**
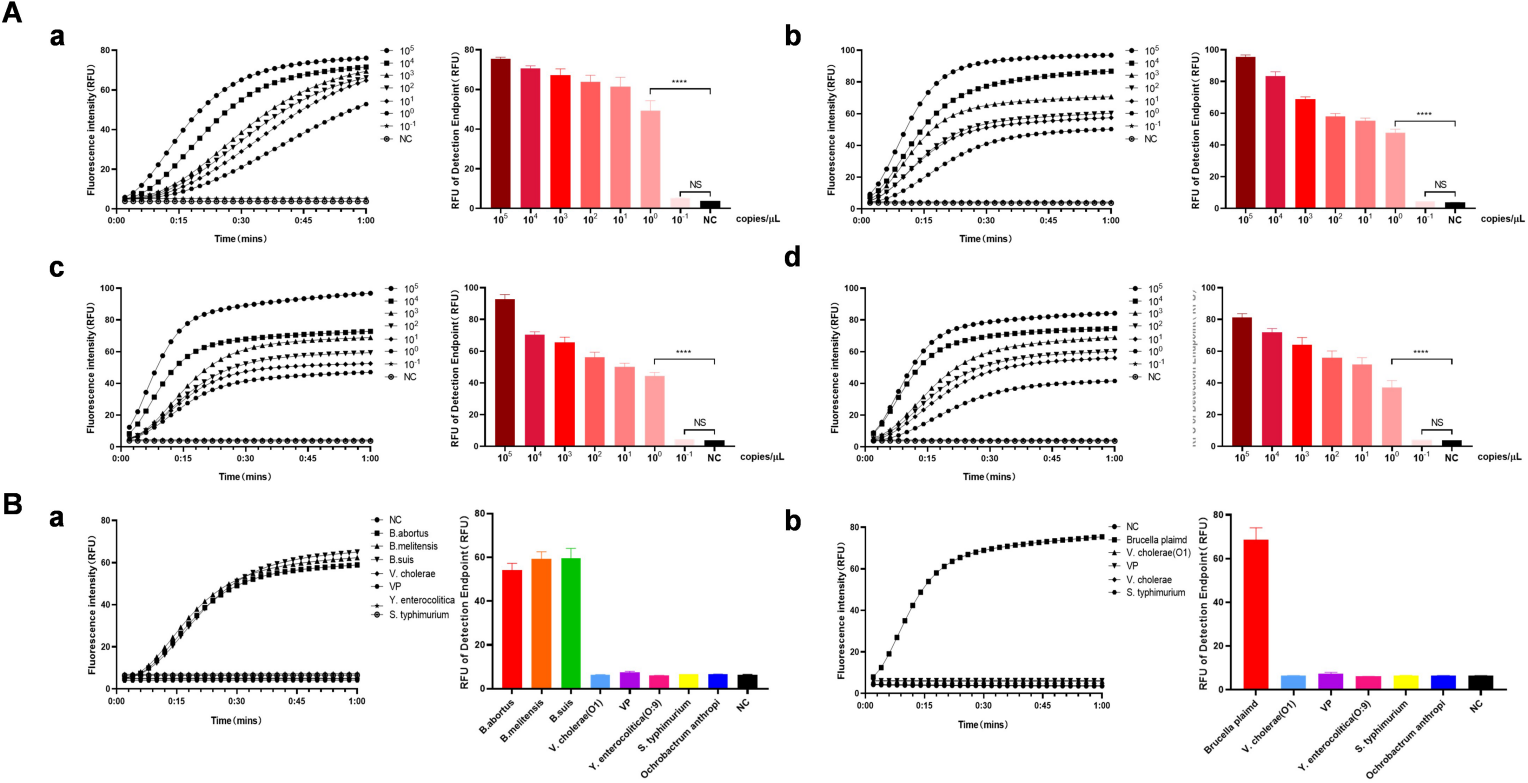
Capability of the Cas13a-crRNA Detection System for Brucella. **(A)** The sensitivity of the fluorescence-based Cas13a-crRNA detection system was assessed using gradient-diluted *Brucella* plasmids **(a)** and *Brucella* standard strains 544A **(b)**, 16M **(c)**, and 1330S **(d)** at concentrations ranging from 10^-1^ to 10^5^ copies/μL. **(B)** The specificity of the Cas13a-crRNA detection system was assessed using both the *Brucella* standard strain **(a)** and a *Brucella* plasmid **(b)** at a concentration of 10^5^ copies/μL.

**Figure 3:**
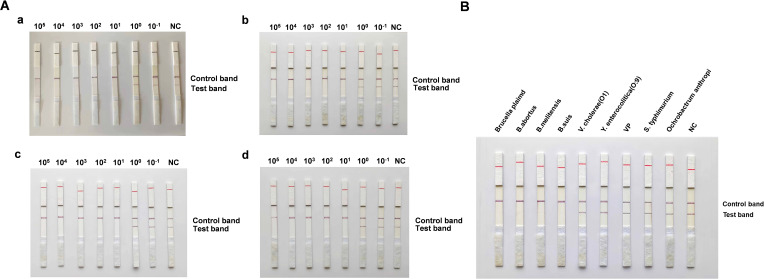
Cas13a-crRNA-Based Strip Assay for *Brucella* DNA **(A)** The sensitivity of the Cas13a-crRNA strip detection system was evaluated using gradient-diluted *Brucella* plasmids **(a)** and standard strains 544A **(b)**, 16M **(c)**, and 1330S **(d)** of *Brucella* (10^-1^ to 10^5^ copies/μL). **(B)** The specificity of the Cas13a-crRNA lateral flow assay (strip detection method) for *Brucella* plasmids was assessed using the *Brucella* standard strains 544A, 16M, and 1330S at a concentration of 10^5^ copies/μL.

The RAA CRISPR/Cas13a *Brucella* fluorescence detection method and the strip detection capability evaluation method were examined for their sensitivity and specificity. The results indicated that the fluorescence signal values for the recombinant positive plasmids of *Brucella* and the RAA amplification products of *B. abortus, B. melitensis*, and *B. suis* biotype I reference strains 544A, 16M, and 1330S, respectively, at a concentration of 10^0^ copies/μL, were significantly higher than those of the negative control (**Figure 2A**). The fluorescence method had the lowest detection limit of 10^0^ copies/μL, while the strip detection method had a detection limit of 10^1^ copies/μL (**Figure 3A**). Validation assays of the fluorescence detection method and the strip test demonstrated that a specific fluorescence curve and positive strip were only obtained for *Brucella* recombinant plasmids and reference strains 544A, 16M, and 1330S of *Brucella* species I in cattle, sheep, and pigs, respectively. In contrast, *V. cholerae, V. parahaemolyticus, Y. enterocolitica, S. typhimurium*, and *B. palustris* did not produce specific fluorescence curves or positive strip results (**Figure 2B**). Further, the established CRISPR fluorescence detection method for *Brucella* exhibited excellent specificity compared to the test strip (**Figure 3B**).

### 3.3 CRISPR assay system for the detection of *Brucella* DNA in milk, blood, and vaginal swab samples

The results indicate that out of 100 milk samples, 80 positive cases were detected with qPCR. This corresponds to a positivity rate of 80%. Further, the positivity rate of ddPCR was 89%; CRISPR fluorescence method, 93%; and CRISPR strip method, 87%. With regard to the human whole blood samples (n = 48), qPCR detected 15 positive cases, which corresponds to a positivity rate of 31.25%. With ddPCR, the positivity rate was 45.83% (22 positive cases). With the CRISPR fluorescence method, the positivity rate was 62.5% (30 positive cases). The CRISPR strip method had a positivity rate of 39.58% (19 positive cases). With regard to the testing of the sheep vaginal swab samples (n = 100), qPCR detected 68 positive cases, corresponding to a positivity rate of 68%. The positivity rate for ddPCR was 84%; the CRISPR fluorescence method, 91%; and the CRISPR strip method, 76% (**Table 1**).

**Table 1:** Sensitivity of qPCR, RAA-CRISPR (F), and RAA-CRISPR (L) for the detection of *Brucella* in 323 actual samples.

	Milk (n = 100)			Human whole blood (n = 123)			Vaginal swabs from sheep (n = 100)		
	Positive	Negative	Sensitivity	Positive	Negative	Sensitivity	Positive	Negative	Sensitivity
qPCR	80	20	80%	74	49	60.16%	68	32	68%
RAA-CRISPR(F)	93	7	93%	101	22	82.12%	91	9	91%
RAA-CRISPR(L)	87	13	87%	79	44	64.23%	76	24	76%

RAA-CRISPR(F), Fluorescence-based CRISPR detection system.

RAA-CRISPR(L), Lateral flow CRISPR detection system.

## 4 Discussion

In this study, we established a lateral flow strip assay for *Brucella* detection based on the RAA-CRISPR/Cas13a system. The minimum detection limit for the fluorescence detection method was 10^0^ copies/μL, and its sensitivity was much higher than that of the ordinary qPCR detection method (91% vs. 68%). With regard to its specificity, the other bacterial species tested were not amplified. This means that the RAA-CRISPR-based fluorescence detection method has high specificity for *Brucella* detection and would be useful in real-life settings. Based on its high sensitivity and good specificity for various types of test samples (including milk, whole blood, and vaginal swab samples), this method can be applied to a variety of sample tests in the future.

The *Brucella* detection system proposed here is based on the CRISPR defense mechanism in bacteria. Ever since the discovery of CRISPR, researchers have been exploring its potential applications in the field of gene editing and nucleic acid detection tools. For example, Zhang Feng’s team reported the development of the “SHERLOCK” detection system based on isothermal amplification, such as RPA combined with CRISPR/Cas13, with which nucleic acid detection was achieved at the level of aM (Gootenberg et al., 2017; Kellner et al., 2020). Such isothermal amplification techniques combined with the CRISPR system have been successfully applied to the detection of a variety of pathogenic bacteria in food, medicine, and clinical practice, especially for the detection of certain pathogenic microorganisms with low load (Tian et al., 2023; Shang et al., 2024). This method often has much higher sensitivity than general molecular biology detection methods and provides good specificity without the need for bulky equipment. In the case of brucellosis, in addition to the sensitivity and specificity of the testing methods, it is important to consider whether the test can be easily applied in the field and in rural areas, which are typically hotspots for this infection. Accordingly, the tool developed in this study is a simple, convenient, and rapid dipstick detection method based on the CRISPR fluorescence assay. One of its advantages is the optimum reaction temperature of 37°C, which is ideal given that the normal human body temperature is also 37°C. Another advantage is the sensitivity of the test strip, which was 10^1^ copies/μL and was higher than that of the qPCR detection method. In addition, the strip showed high specificity for *Brucella* samples, as it only tested positive for the *Brucella*-containing samples and was negative for the remaining pathogens (*V. cholerae*, *V. parahaemolyticus*, *Y. enterocolitica*, *S. typhimurium*, and *Ochrobactrum anthropi*. These observations could be replicated in actual sample detection tests, too, as the constructed dipstick showed good sensitivity and specificity for different types of samples (whole blood, milk, and vaginal swabs). Importantly, compared with qPCR and ddPCR, the RAA-CRISPR/Cas13a *Brucella* strip detection method established in this study comes at a lower cost while offering higher efficiency, and does not require bulky equipment. More specifically, the minimum detection limit is higher than that of the conventional qPCR assay, and the detection time is greatly shortened to 45 min. Overall, this method has the advantages of being simple, rapid, efficient, highly sensitive, and specific, and greatly reduces the cost of detection. Moreover, it can be applied in a variety of complex environments for the real-time, efficient detection of *Brucella* and could be particularly useful for the rapid visual detection of *Brucella* and preliminary screening for brucellosis in resource-limited environments. In the future, this method is likely to be widely applied to the rapid screening of *Brucella* by grassroots medical and health institutions.

## Data Availability

The original contributions presented in the study are included in the article/**Supplementary Material**. Further inquiries can be directed to the corresponding authors.
