# Supplementary material for: A CRISPR/cas13a-assisted precise and portable test for Brucella nucleic acid detection
Source: Front Cell Infect Microbiol. 2025 Mar 21;15:1545953. doi: 10.3389/fcimb.2025.1545953 (PMC11968652; doi:10.3389/fcimb.2025.1545953)
Supplement: Supplementary file 1 [file Table1.docx]

| **Name** | **Sequences** |
| --- | --- |
| F1 | 5’-AATTCTAATACGACTCACTATAGGG  TAACGGCACCGGCCTTTATGATGGCAAGGG-3’ |
| R1 | 5’-CGCGCTTGCCTTTCAGGTCTGCGACCGATT-3’ |
| F2 | 5’-AATTCTAATACGACTCACTATAGGG  CTATTGGGCCTATAACGGCACCGGCCTTTA-3’ |
| R2 | 5’-TCAGGTCTGCGACCGATTTGATGTTTGCAT-3’ |
| F3 | 5’-AATTCTAATACGACTCACTATAGGG  TTGCCTATTGGGCCTATAACGGCACCGGCC-3’ |
| R3 | 5’-GTCTGCGACCGATTTGATGTTTGCATCCTT-3’ |
| F4 | 5’-AATTCTAATACGACTCACTATAGGG  CCTATAACGGCACCGGCCTTTATGATGGCA-3’ |
| R4 | 5’-CTTGCCTTTCAGGTCTGCGACCGATTTGAT-3’ |

Supplementary Material

# Supplementary Table1. Sequences of Brucella DNA RAA primers

T7 promoter sequences are colored in red.

# Supplementary Table2. CRISPR/Cas13a-related crRNA sequences

| **Name** | **Sequences** |
| --- | --- |
| *Brucella*-crRNA-1 | GGGATTTAGACTACCCCAAAAACGAAGGGGACTAAAAC  GAAGGCGCAAATCTTCCACCTTGCCCTT |
| R1 | AAGGGCAAGGTGGAAGATTT |
| *Brucella*-crRNA-2 | GGGATTTAGACTACCCCAAAAACGAAGGGGACTAAAAC  GTCGCCAGAAGGCGCAAATCTTCCACCT |
| R2 | AGGTGGAAGATTTGCGCCTT |
| *Brucella*-crRNA-3 | GGGATTTAGACTACCCCAAAAACGAAGGGGACTAAAAC  GTAAAGCGTCGCCAGAAGGCGCAAATCT |
| R3 | AGATTTGCGCCTTCTGGCGA |
| *Brucella*-crRNA-4 | GGGATTTAGACTACCCCAAAAACGAAGGGGACTAAAAC  CGCAACGATATGGATCGTTTCCGGGTAA |
| R4 | TTACCCGGAAACGATCCATA |
| *Brucella*-crRNA-5 | GGGATTTAGACTACCCCAAAAACGAAGGGGACTAAAAC  GTTTCCGGGTAAAGCGTCGCCAGAAGGC |
| R5 | GCCTTCTGGCGACGCTTTAC |
| T7-crRNA-F | TAATACGACTCACTATAGGGGATTTAGACTACCCCAA |
